# A bankfull geometry dataset for major exorheic rivers on the Qinghai-Tibet Plateau

**DOI:** 10.1038/s41597-022-01614-w

**Published:** 2022-08-16

**Authors:** Dan Li, Yuan Xue, Chao Qin, Baosheng Wu, Bowei Chen, Ge Wang

**Affiliations:** 1 Emergency Science Research Institute, Chinese Institute of Coal Science, Beijing, 100013 China; 2 State Key Laboratory of Hydroscience and Engineering, Department of Hydraulic Engineering, Tsinghua University, Beijing, 100084 China; 3 Key Laboratory of Digital Earth Science, Aerospace Information Research Institute, Chinese Academy of Sciences, Beijing, 100094 China

**Keywords:** Hydrology, Environmental impact

## Abstract

Bankfull river discharge shapes river morphology. The bankfull river surface planform and river width can be used to quantify river size. Regional studies of stream ecology, hydrologic modelling, river carbon emissions and geomorphology from the perspective of fluvial processes are hindered by the lack of a highly accurate spatially distributed river network that considers bankfull river geometry. Based on Sentinel-2 and Landsat 5/7/8 multispectral instrument imagery and *in situ* measured hydrological data, the river discharge and width of spatially distributed cross sections of six major exorheic rivers and their tributaries located on the Qinghai-Tibet Plateau (QTP) are calculated under bankfull conditions. Then, the bankfull river surface is extracted. Finally, a bankfull river width and surface area database is established. The provided planform river hydromorphology data can supplement global hydrography datasets and effectively represent the combined fluvial geomorphology and geological background in the study area.

## Abstract (summary)

**Table:** 

Measurement(s)	bankfull river width and surface area
Technology Type(s)	remote sensing
Factor Type(s)	discharge
Sample Characteristic - Environment	rivers on plateau
Sample Characteristic - Location	Qinghai-Tibet Plateau

## Background & Summary

Bankfull discharge (BD) plays a dominant role in shaping river morphology, as it is associated with the transition from shaping channels to shaping floodplains with variations in the flow and sediment load. In alluvial rivers, BD is geomorphically important because it generally represents the discharge that influences channel geometry (width and depth), and it is also called channel-forming discharge^1^. BD is widely used in river management and restoration because cross-sections shaped by BD often effectively convey water and sediments. The frequency with which a river experiences BD conditions varies from several times a year in humid environments to once every several years in semiarid regions^1^. River surfaces are interfaces for a host of mass and energy exchange processes with the atmosphere and biosphere^2^, and the corresponding bankfull geometries reflect comprehensive fluvial processes. Therefore, studies of the dynamics of BD and the corresponding river surface planform at fine spatial resolutions can support further research on fluvial geomorphology and river sediment processes, and the obtained information can be applied in stream carbon emission estimation, ecological assessments, river restoration, hydrological modelling, and water resources management, among others^3^.

The Qinghai-Tibet Plateau (QTP), known as Asia’s Water Tower and the Earth’s third-largest ice reservoir, is the origin of 10 major rivers that flow through the Asian continent^4^. This region includes a wide range of climates and underlying surfaces, which develop a wide variety of river patterns from single-thread to multi-thread and from rock-constrained to free-flowing^5^. The complex background environment leads to various fluvial geomorphologies, which can be represented by the distributions of bankfull river widths and surface planforms across river networks.

Currently, many global and regional datasets are maintained for mountain river networks on the QTP. Data are derived from digital elevation models (DEMs) or remote sensing imagery. The most widely used, publicly available global hydrography dataset, HydroSHEDs, is DEM-derived and includes drainage networks (HydroRIVERs) for the whole QTP^6^. However, the constant threshold (100 pixels for DEM rasters) of flow accumulation may result in the incorrect generation of river networks (e.g., misidentifying permanent gullies as small rivers). Follow-up products have resolved this issue by using variable drainage areas and slope gradients as thresholds in detecting river networks and yielded more accurate results^7–9^. However, those products are fully DEM-derived and have not been validated by field observations or remote sensing imagery at the regional scale. To what extent they represent the real river (but not gully) network has been questioned. Moreover, the above products lack river geometry information, which restricts the ability to obtain geomorphological information from the rivers^10^.

Remote sensing (RS) is an effective method for extracting the surface of water bodies over a variety of spatiotemporal scales compared with other field survey methods employed in recent decades^3^. River networks generated from imagery encompass the appropriate perennial drainage. Based on Landsat 5/7/8 images, the global water surface database^11^ provides the basic conditions for the QTP water surfaces of rivers, lakes and reservoirs. The Global River Widths from Landsat (GRWL) database^2^, including width information under mean annual discharge conditions, presents a basic characterization of the rivers on the QTP. However, no regional bankfull river geometry dataset (BGD) has been launched thus far due to the scarcity of *in situ* measured hydrological data, the low frequency of BD events and the limited availability of remote sensing imagery. In addition, rivers with widths <90 m are not fully and correctly characterized, even under mean annual discharge^2,12^; thus, physical meaning is lacking for some fluvial processes, e.g., changes in channel morphology.

In summary, limitations of the existing river network databases include (1) lack of river width information, (2) lack of a complete characterization of small rivers with widths <90 m, and (3) lack of clear physical meaning. In this study, Sentinel-2 imagery and Landsat 5/7/8 imagery were used to extract river surfaces under bankfull conditions on the QTP. Three products are provided: a bankfull river surface dataset, a bankfull river width dataset and a bankfull river surface area dataset. These datasets provide a fundamental basis for various studies on the QTP, the third pole of the world, including studies on geological geomorphology^13^, hydroinformatics^14^, biogeochemical cycles^15^, plateau ecology, etc.

## Methods

### Data used in this study

The data used in this study include (1) satellite imagery data (Sentinel-2 Level-1C (L1C) and Landsat 5/7/8 Tier 1 (T1) surface reflectance (SR)), (2) QTP boundary data, (3) QTP DEM data, (4) QTP river network data, and (5) *in situ* measured hydrological data. The Sentinel-2 L1C and Landsat 5/7/8 T1_SR data were obtained from the GEE cloud platform by ee.ImageCollection(). Details of the data used in this study are introduced in Table 1. Abbreviations for the six exorheic rivers are: YR (Yellow River), JSR (Jinsha River), YLR (Yalong River), NR (Nu River), LCR (Lantsang River), YLZBR (Yarlung Zangbo River).

**Table 1:** Details of the data used in this study.

Data	Sources	Time Period	Instructions	Basins for which the data were used
Qinghai-Tibet Plateau boundary^31^	http://www.tpdc.ac.cn/en/data/61701a2b-31e5-41bf-b0a3-607c2a9bd3b3/	2016	The dataset contains five types of boundaries, and TPBoundary_HF was used.	YR, JSR, YLR, NR, LCR, YLZBR
ASTER Global Digital Elevation Model, Version 3	https://earthdata.nasa.gov/learn/articles/new-aster-gdem	2019	30 m resolution; used for contributing region generation of 23 cross-sections	YR, JSR, YLR, NR, LCR, YLZBR
HydroSHEDs river network^18^	https://www.hydrosheds.org/page/hydrorivers	2008	Rivers are classified into 8 orders	YR, JSR, YLR, NR, LCR, YLZBR
*In situ* measured Hydrological data (river width, flow discharge, and cross-section morphology)	Annual Hydrological Reports of P. R. China (http://www.mwr.gov.cn/english/)	1967–2020	Data from 23 cross-sections across the QTP were used for BD calculations	YR, JSR, YLR, NR, LCR, YLZBR
Sentinel-2	GEE cloud platform (https://code.earthengine.google.com/)	2017–2020	10 and 20 m spatial resolutions; 5-day revisit period; 13 bands	YR, JSR, YLR
Landsat-5	GEE cloud platform (https://code.earthengine.google.com/)	1984–2011	30 m spatial resolution; 16-day revisit period; 7 bands	NR, LCR, YLZBR
Landsat-7	GEE cloud platform (https://code.earthengine.google.com/)	1999–2020	30 m spatial resolution; 16-day revisit period; 8 bands	NR, LCR, YLZBR
Landsat-8	GEE cloud platform (https://code.earthengine.google.com/)	2013–2020	30 m spatial resolution; 16-day revisit period; 11 bands	NR, LCR, YLZBR

The codes used in this study include (1) JavaScript algorithms used for cloud-free imagery composition and water surface extraction and (2) MATLAB algorithms used for the generation of skeletonized river networks, with control points every 1 km along river channels and cross sections for the skeletonized river networks. The algorithms are introduced in “Code Availability”.

**Fig. 1:**
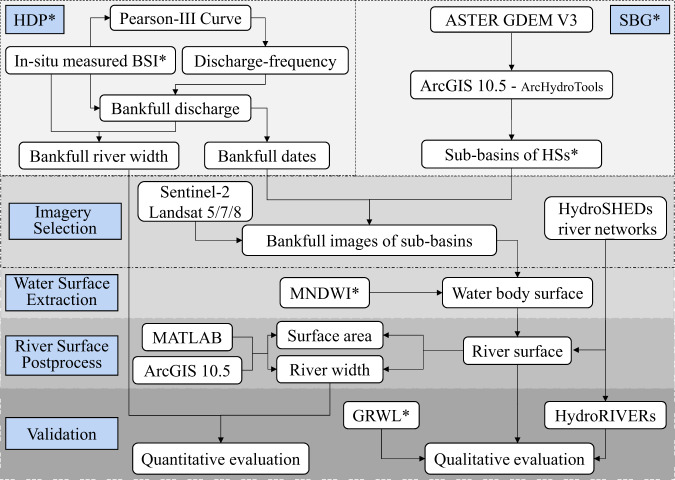
Technical flow chart of this study.

The overall procedure consisted of six steps: hydrological data preprocessing (HDP), contributing regions generation from an ASTER Global DEM (GDEM, Version 3) (SBG), remote sensing imagery selection under BDs, water surface extraction, river surface postprocessing, and validation, as shown in Fig. 1. We used the Google Earth Engine (GEE), ArcGIS 10.5 (ESRI Inc., Redlands, CA, USA), and MATLAB R2017b (Mathworks Inc., Massachusetts, USA) to implement the research procedure.

### Hydrological data preprocessing

#### *In-situ* measured data screening

**Fig. 2:**
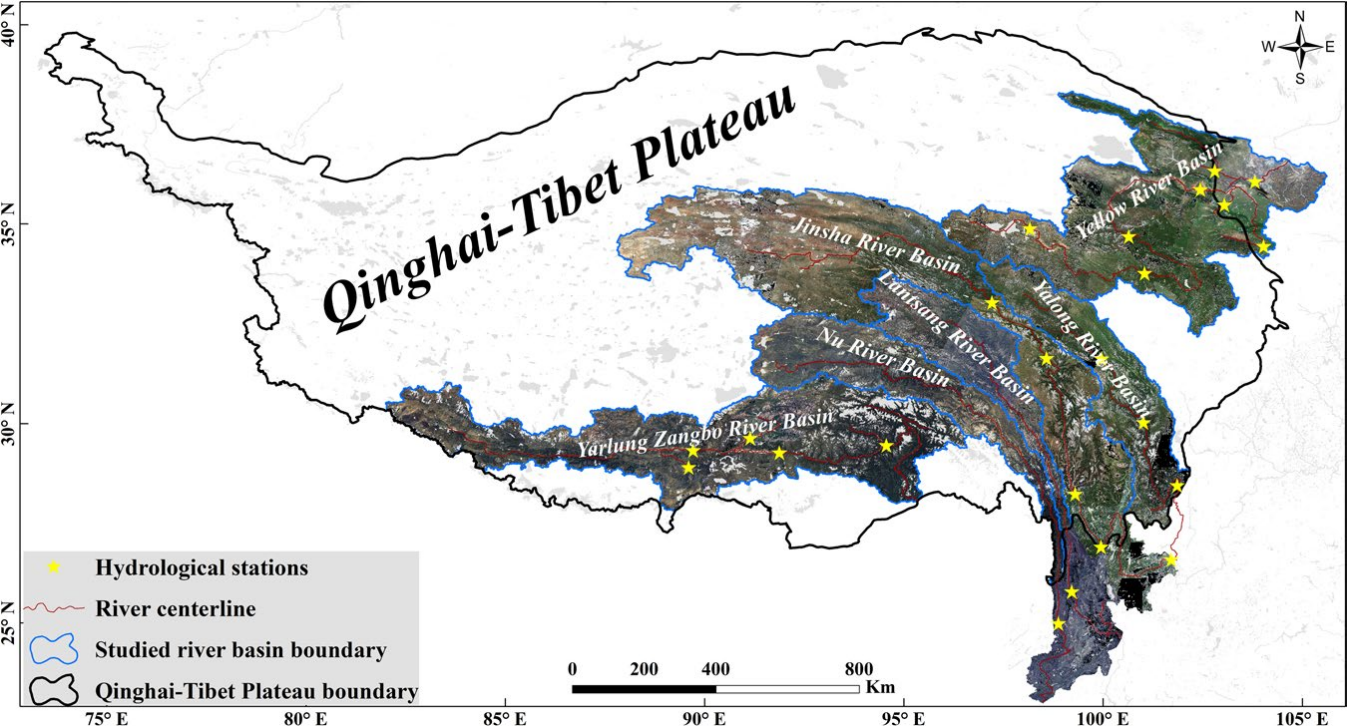
The study region, remote sensing imagery coverage, and 23 *in situ* measured cross sections located at national hydrological stations in the studied river basins and used for image selection.

The *in-situ* measured river width, flow discharge in cross sections and section morphologies used for BD calculations should meet the following criteria: (1) obtained at national hydrological stations with a consecutive hydrological record length exceeding 10 years; (2) relatively low anthropogenic influence (e.g., no hydropower stations or artificial diversion structures 10 km upstream and downstream of the measured cross section; located outside the backwater zone of a dam) during the study time period to minimize the external disturbance; (3) located away from regions that might be affected by extreme events such as glacial outbursts, landslides, etc.; and (4) sampled water body is a natural riverway with perennial drainage^5^. After initial filtering based on the above four criteria, 58 cross sections were retained. Then, comprehensively considering the spatial distribution and contributing areas of the cross-sections and the relations between the mainstream and tributaries, 23 cross-sections were used in the subsequent imagery selection process and calculations of water level, river width and discharge under bankfull conditions (Fig. 2 and Supplementary File 1 Table 1 available at Figshare). The cross-sections located in mainstreams were distributed uniformly in the upper, middle and lower parts of watersheds. For the same tributary, at most one cross-section was used for imagery selection.

#### Calculation of discharge and river width under bankfull conditions

Annual peak discharge values (no less than 20 years) at the 23 selected cross sections were recorded according to the Annual Hydrological Reports of the P. R. China. The detailed steps for bankfull discharge and width calculations are as follows.

1. Determination based on cross section morphology
  The morphologies of 23 cross-sections were first visualized to detect turning points (indicating the connections between floodplains and main channels). The potential bankfull position of each cross section was recorded if an evident break was observed in the cross section morphology curve^16^. Supplementary field investigations were conducted to identify the turning points based on real bankfull positions. BD, the corresponding water level, and river width were then determined considering the associated upstream and downstream relationships. For instance, if there are 5 cross-sections in the same river reach and the water levels and discharge values under bankfull conditions can be determined based on cross-section morphology, then the BDs at the other 2 cross-sections can be approximated based on the mean of the three known cross-sections. Finally, the discharge frequency corresponding to the potential bankfull position was checked to see whether it met the relevant general requirements. All 23 cross-sections have a recurrence interval of less than 8 years and were therefore used in the following analysis^17^. BD values that could not be determined from the cross-section morphology were estimated with the following method.
2. In assessments of cross-sections without evident floodplains (as described above), the following factors were considered: stream order, contributing area, upstream and downstream relationships, and mainstream and tributary relationships. The main processes were as follows: (1) determine the mean of known BD frequencies in the same river reach as the BD frequency for unknown cross-sections; (2) if there is no known BD frequency in the same reach, use the BD frequency for a stream of the same order as a substitute; (3) check the BD frequencies determined in the above two steps using the Pearson III discharge-frequency curve; and (4) the estimation is reliable if the BD increases from upstream to downstream; if not, check whether the discharge increases downstream and the cross-section is influenced by evident anthropogenic disturbances, such as water divisions or reservoirs. The determined discharge and river widths under bankfull conditions for 23 cross-sections are presented in Supplementary File 1 Table 2 at Figshare.

### River networks and the extraction of contributing regions

The HydroSHEDs river network dataset^18^ was chosen as the basis to build buffer zones to constrain RS images (Table 1). The contributing region of each hydrological station was generated using hydrological station coordinates and ASTER GDEM V3. Standard processes for hydrological analysis were implemented with ArcHydroTools in ArcGIS 10.5.

### RS imagery selection

#### Criteria

RS imagery was selected according to two sets of criteria: (1) Sentinel-2 MSI imagery was the priority data source for extracting the water surface if the coverage was complete on the selected dates, and (2) a less-than-ideal alternative, Landsat TM/ETM + /OLI imagery, was selected if the coverage of the Sentinel-2 MSI imagery was not complete on certain dates.

#### Processes

The detailed imagery selection processes were as follows: (1) BD and the corresponding dates (date/month/year) for each cross-section were recorded; (2) the availability of Sentinel-2 MSI images that could completely cover the target area on the selected dates was checked; and (3) for cross-sections with incomplete imagery coverage or large amounts of cloud cover, the ratio of daily discharge (DD) to BD was expanded by at most ±50% to increase the image coverage. For instance, the DD ranges of the upper YR, the upper JSR and the YLR were ±10% to ±20%, ±20%, and ±40% to ±50%, respectively (Supplementary File 1 Table 2 available at Figshare); (4) for rivers that were not completely covered by Sentinel-2 MSI images, even with a ±50% DD, Landsat images were then used as an alternative data source (Table 1); (5) the above steps were repeated to obtain complete Landsat imagery coverage.

### RS imagery preprocessing

#### Imagery cloud-free composition

Cloud is an important atmospheric component that affects water body extraction. We used the ‘QA60’ band to create a cloud mask for the Sentinel-2 imagery. For Landsat imagery, we referred to the method developed by Housman *et al*.^19^ and used the ‘landsatCloudScore’ function to compute a cloud score for the cloud mask. All operations were performed on the GEE platform, and the JavaScript algorithms are available in Supplementary File 2 at Figshare (River extraction from Landsat.js and River extraction from Sentinel 2.js).

After the above selection and processing steps, Sentinel-2 images were used for the three northeastern river basins (YR, JSR, and YLR), and Landsat 5/7/8 images were used for the river basins (NR, LCR, and YLZBR) located in the southern part of the QTP (Table 1). The remote sensing imagery coverage is shown in Fig. 2.

#### Image clipping

Background noise (e.g., complex terrain, vegetation, clouds, snow and hill shadows) considerably impacts river extraction. To minimize the influence of background noise and improve the efficiency of river extraction, we used the HydroSHEDs river networks to build buffer zones. Due to the positional deviation between the HydroSHEDs river networks and the real river centerlines extracted from RS images, we built the buffer zones with different buffer radius for different Horton-Strahler stream-order reaches.

Specifically, based on the measured river widths from hydrological data and Google Earth images, the buffer radius of order 3–4 rivers was set to 1000 m, the buffer radius of order 5–6 rivers was set to 2500 m, and the buffer radius of order 7–8 rivers was set to 3500 m. RS images under bankfull conditions inside the buffer zones were selected for river extraction. These improvements could maximally reduce the workload of artificial editing, allowing the proposed method to be applied over large areas.

### Water surface extraction and postprocessing

#### Water surface extraction

We used the modified normalized difference water index (MNDWI) constructed by Xu^20^ to extract water bodies from Sentinel–2 and Landsat 5/7/8 satellite imagery based on the GEE cloud platform:

$$MNDWI=\frac{green-swir1}{green+swir1}$$

where *green* refers to Sentinel-2 band 3 or Landsat 5/7/8 band 2 and *swir*1 refers to Sentinel-2 band 11, Landsat 5/7 band 5 or Landsat-8 band 6. The spatial resolution of Sentinel-2 was uniformly resampled to 10 m.

An appropriate threshold to differentiate between water and nonwater pixels can significantly reduce omission and commission errors in spectral water indices^21^. One key purpose of utilizing water indices to extract water bodies is to simplify image classification by defining the zero-water index value as the threshold for differentiating water and nonwater pixels. However, this single zero-water index threshold method may not work well due to spatiotemporal changes in brightness and contrast in remote sensing images^22^. The terrain and underlying surface of the study area are complex, which may result in poor extraction performance when a fixed threshold is used.

For Sentinel-2 images, we used the MNDWI with Otsu thresholding to separate water from nonwater objects in the buffer zones generated based on the HydroSHEDs river networks under bankfull conditions. Otsu’s method was designed to distinguish between the background and foreground in images by creating two classes with minimal intraclass variance^23^. This approach uses a maximum between-class variance criterion to determine the optimal threshold for image segmentation^24^. We used Otsu’s method to determine the initial threshold of the MNDWI and then manually adjusted the dynamic threshold by referring to the histogram of MNDWI values. Different MNDWI thresholds were used for the contributing regions of different hydrological stations to minimize the influence of shadows, snow and vegetation on the water body results. The optimal thresholds of the MNDWI in different contributing regions were determined by visual inspection based on the principles of minimizing background noise and optimizing river extraction.

For Landsat 5/7/8 images, we used the zero-MNDWI threshold value to extract water bodies. Then, we referred to the method developed by Donchyts^25^. The blue band and thermal infrared temperature band were used to minimize the influence of shadows and snow to improve the accuracy of water body extraction. The relevant JavaScript algorithms used in this step are provided in Supplementary File 2 at Figshare.

#### River surface extraction

**Fig. 3:**
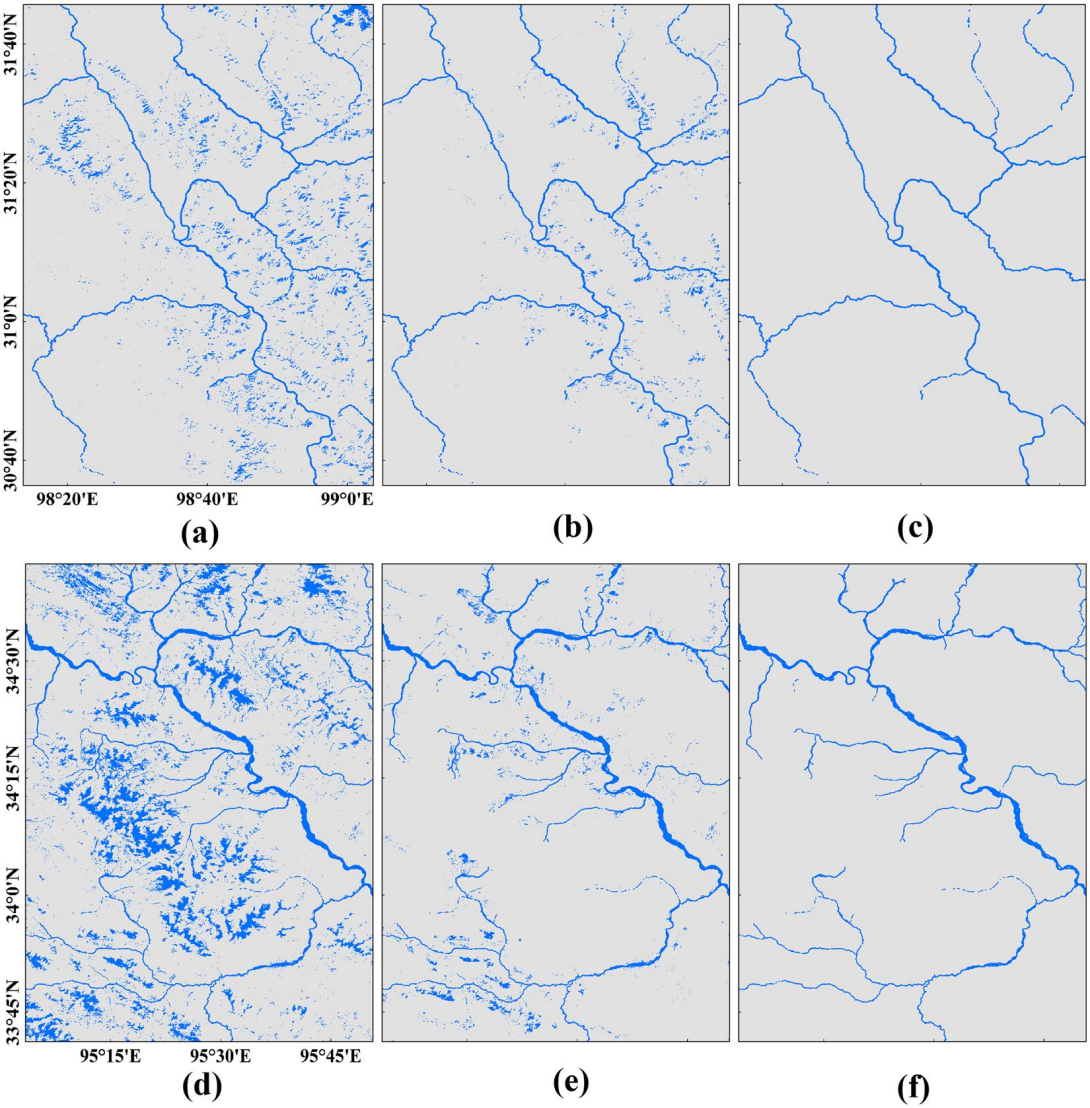
Results of the denoising and artificial editing of water surfaces located in the JSR Basin. (**a**,**d**) Original water surface results extracted from remote sensing imagery; (**b**,**e**) water surface results after the HydroSHEDs river network constraint was applied; and (**c**,**f**) final river surface results after denoising and artificial editing.

Due to the effects of bridges, clouds and shadows, there were gaps in the river extraction results. We artificially connected these gaps in high-order river reaches and filled the gaps associated with shoals, central bars, etc., using topology processing in ArcGIS 10.5. Then, we artificially removed other noise inside the buffer zones to obtain the final bankfull river surface product. The river width was defined as the farthest distance between the left and right sides of a river (Fig. 6c of Li *et al*.^12^). Two types of rivers were included in the BGD: connected rivers and disconnected rivers. The postprocessing procedure is shown in Fig. 3.

### River width extraction and river surface area calculation

#### Extraction of river centerlines

Based on the bankfull river surface dataset (connected rivers) obtained in the above section, we developed an automated river width and surface area extraction procedure under the MATLAB and ArcGIS 10.5 framework. The main steps include the extraction of river centerlines, river widths and river surface areas.

**Fig. 4:**
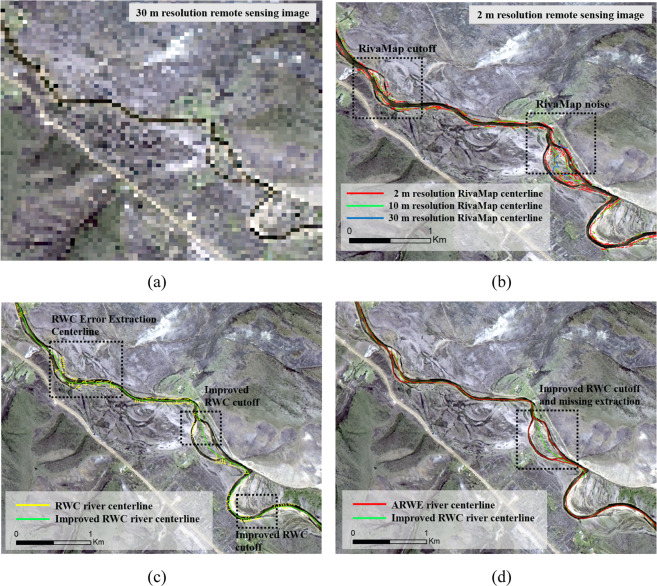
River centerlines extracted by the RivaMap, RWC and ARWE methods in a mountain river reach located in the Jimai River basin (the first order tributary of the upper Yellow River): (**a**) Landsat imagery (30-m resolution) of the mainstream of the Jimai River. (**b**) River centerlines extracted by the RivaMap method from 2 m-, 10 m- and 30 m-resolution remote sensing images using the multiscale singularity index, which was adjusted based on the method recommended by Isikdogan *et al*.^28^. (**c**) River centerlines extracted by the RWC method using the gradient thresholds recommended by Yang *et al*.^27^ and an improved RWC method, in which the 9*9 kernels used to convolve the binarized river centerlines and the river centerlines scope were determined by unsupervised method. (**d**) River centerlines extracted by the ARWE and the improved RWC. The geographical location of the Jimai River is presented in Fig. 6(a).

Existing methods, including the RivWidth algorithm^26^, RivWidthCloud algorithm (RWC)^27^ and RivaMap^28^, have some defects in extracting the centerlines of small mountain rivers located on the QTP (Fig. 4, Supplementary File 1 Table 3 available at Figshare). Generally, two sides of a mountain river do not display strict symmetry, which results in the river centerlines obtained by these methods exhibiting evident offsets, especially in sections with sharp river bends. Thus, an enormous manual editing effort is needed. In addition, these methods require artificial supervision and a large number of global convolution operations, even though they are automated. For instance, the RWC algorithm obtains the distance gradient of each pixel by performing multiple convolution operations on binarized river surface data and then manually sets the gradient discrimination threshold to obtain the corresponding centerline. Therefore, we developed the Automated River Width Extraction (ARWE) method, which can automatically correct the river skeleton lines to the river centerlines. This method does not require convolution operations for all pixels multiple times. Detailed steps for river centerlines extraction from the river surface with the ARWE method are as follows:

1. The river framework was extracted from the river surface with the skeleton algorithm and used to define the river pseudocenterlines after being encoded.
2. The intersection between the orthogonal river pseudocenterlines and the river boundaries extracted by the morphological edge detection algorithm were calculated. A line that crossed intersection points was considered a pseudoriver cross-section.
3. The pseudoriver cross-sections were encoded with the same code that was used for the pseudocenterlines.
4. The midpoints of each pseudoriver cross-section were calculated and encoded. The code of each midpoint was recorded as the pseudoriver width section. The midpoints were connected in the order of encoding to obtain the initial corrected river pseudocenterlines.
5. Steps (3–4) were repeated until the distance between the midpoints in two adjacent calculations for cases with the same code number was less than or equal to the image resolution. Connected the last calculated midpoints in the order of encoding to obtain the ARWE river centerlines result. A continuous raster river framework with a width of one pixel was extracted. Steps (1–6) are performed in MATLAB, and the algorithms are available in Supplementary File 3 at Figshare (ARWE_CompCode.m).

#### River width extraction

1. Converted the raster skeleton lines into uninterrupted vector lines in ArcGIS 10.5.
2. According to the river scale and stream order, 1 km was set as the river length interval for width extraction. Then, marked the control points in the skeletonized river networks with a 1 km length interval. This step was completed in ArcGIS 10.5.
3. Drew perpendicular lines from the control points to the left and right sides of the river networks. These control points were the virtual hydrological stations where river widths was calculated. This step is completed in MATLAB, and the algorithms are available in Supplementary File 3 at Figshare.
4. The algorithm searched for the boundary points of riverbanks from each control point along the perpendiculars. When the search process reached the preset maximum value, the detection procedure stoped, and the paired boundary points were marked. This step is completed in MATLAB, and the algorithms are available in Supplementary File 3 at Figshare.
5. The length of the line connecting the two boundary points was recorded as the river width every 1 km and exported from ArcGIS 10.5.

The steps in river width extraction and the applied concepts are presented in Supplementary File 1 Fig. 1 at Figshare.

#### Calculation of river surface area

Each river surface was clipped according to the central perpendiculars, and the river surface area was calculated for every 1 km river length. This step was completed in ArcGIS 10.5.

### Comparison of the existing methods for extracting river width

We tested three widely used river width extraction methods, namely, RivWidth^26^, RivaMap^28^ and RivWidthCloud^27^, and ARWE with data from one of the first tributaries of the Yellow River Basin. The advantages and disadvantages of the above three methods are compared in Supplementary File 1 Table 3 at Figshare. In detail, the RivWidth algorithm obtains the boundary of a river based on the river surface, uses a large number of vertical lines perpendicular to the boundary that intersect the river channel, and obtains centerlines through the intersection of the vertical lines. The RivWidthCloud algorithm obtains the distance gradient in each pixel by performing multiple convolution operations on the binarized river surface data and then artificially sets a gradient discrimination threshold to obtain the corresponding centerlines. Although these methods are automatic, they require artificial supervision and a large number of global convolution operations. RivaMap uses a quasi-real-time water surface extraction algorithm based on preset MNDWI values and pretreated RS images. However, artificial judgement of the MNDWI is needed during the pretreatment of RS images, and the empirical determination of the singularity index range is required.

With the Jimai River (first-order tributary of the upper Yellow River) as an example, we extracted river centerlines using RivaMap, RivWidthCloud and ARWE. The results are shown in Fig. 4. Specifically, we extracted river centerlines using the RivaMap algorithm based on RS images with three resolutions (2, 10 and 30 m). The multiscale singularity indices were adjusted based on the method recommended by Isikdogan *et al*.^28^. The noise level increased with increasing of texture information, and this issue might lead to poor results in river centerline extraction when using high-resolution RS imagery. The RWC river centerlines have more discontinuities than the ARWE river centerlines. Many parameters used for empirical-based determination, such as the direction of the river centerline in RivWidth, the gradient discrimination threshold in RivWidthCloud, and the singularity index range in RivaMap, should be improved to be less dependent on human judgement.

Overall, the ARWE method improves the accuracy of extracted mountain river centrelines. This method is applicable in both single-thread and multi-thread river reaches, especially for small mountain rivers. Many parameters used in empirical-based determination, such as the direction of the river centerline in RivWidth, the gradient discrimination threshold in RivWidthCloud, and the singularity index range in RivaMap, decrease the applicability of these methods in cases involving mountain rivers extraction. The unsupervised central axis transformation algorithm proposed in our research is embedded in the ARWE method, thus minimizing the influence of human factors on river centerlines extraction. In addition, river widths extracted from the ARWE centerlines are more accurate than those extracted from DEM river networks, as were previously used^3^.

## Data Records

Three products are included in the dataset^29^: one original product (bankfull river surface dataset) and two derived products (bankfull river width dataset and bankfull river surface area dataset with a 1 km river length interval). These three products are available at https://figshare.com/ and are in three folders. The first folder, “1-Bankfull River Surface”, contains river surface vectors for six river basins in the.shp file. The second folder, “2-Bankfull River Width”, contains bankfull river widths and corresponding coordinates with a 1 km-step river length for six mainstreams and some connected tributaries in.xlsx format. The river width vectors in the.shp files are also provided in the second folder. The third folder, “3-Bankfull River Surface Area”, contains bankfull river surface areas and corresponding coordinates with a 1 km-step river length for six mainstreams and some connected tributaries in.xlsx format. Individual river surface vectors for every 1 km river length in the.shp files are also provided in the third folder. Detailed information on the contents of the three generated datasets, data types, and other remarks is presented in Table 2.

**Table 2:** Basic information on the three bankfull datasets (river surface, river width and river surface area).

Dataset	Data type	Contents	Remarks
Bankfull river surface data	vector data	connected and disconnected bankfull river surface planform	can be used for width and surface area calculations at any river length step
Bankfull river width data	vector and excel data	bankfull widths of connected rivers and the corresponding coordinates along with HYRIV_IDs	every 1 km along the connected river length
Bankfull river surface area data	vector and excel data	bankfull surface areas of connected rivers and the corresponding coordinates	every 1 km along the connected river length

## Technical Validation

**Fig. 5:**
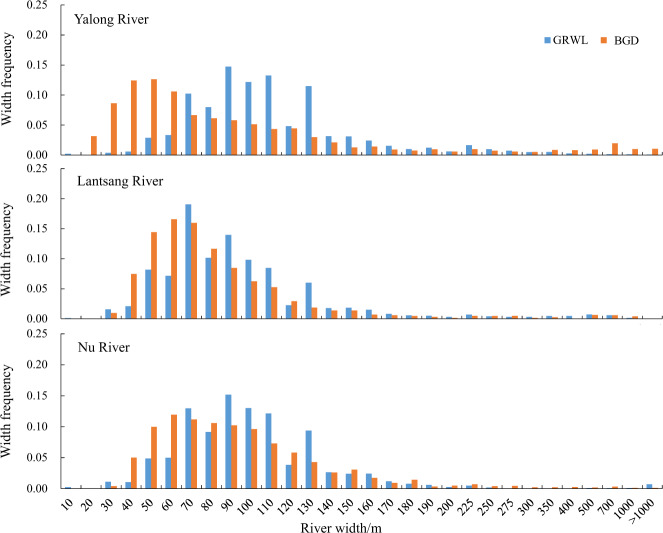
Frequency distributions of river widths for the Yalong River, Lantsang River and Nu River based on the BGD and GRWL database.

**Fig. 6:**
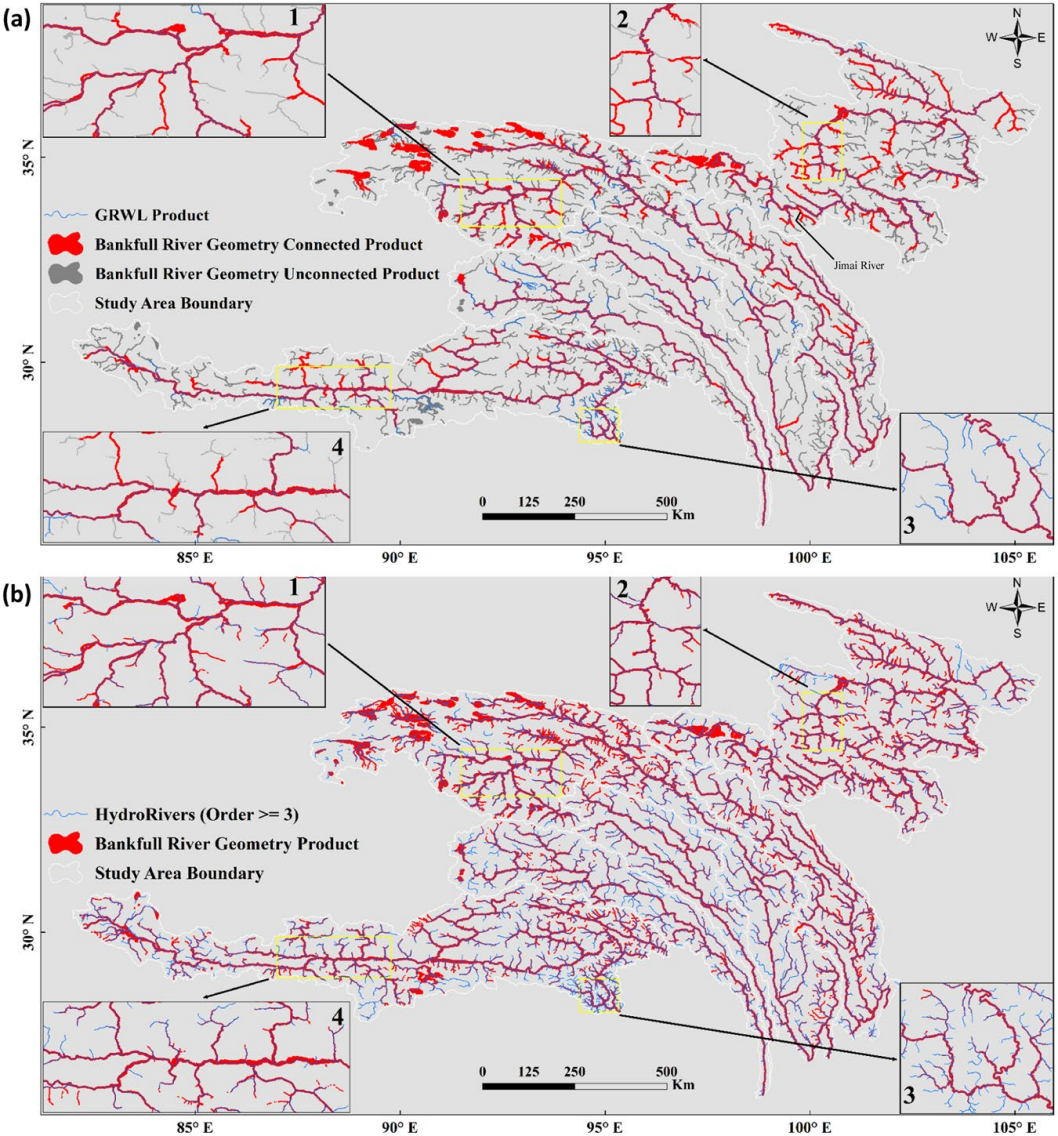
Comparison of the BGD and existing databases ((**a**) GRWL and (**b**) HydroSHEDs).

**Fig. 7:**
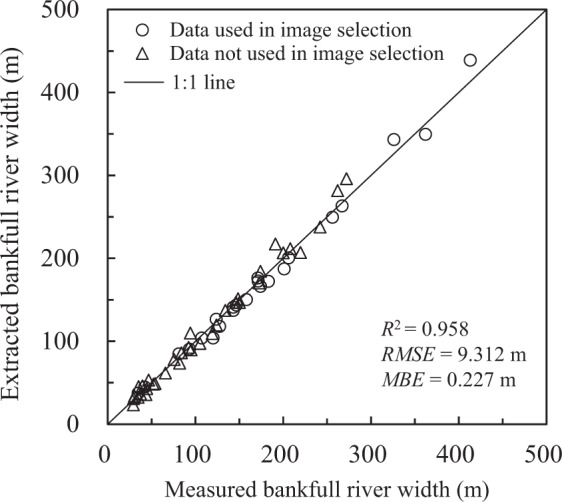
Bankfull river widths derived from Sentinel-2 and Landsat 5/7/8 imagery compared with corresponding *in situ* measured bankfull river widths.

As it has been demonstrated that small rivers with widths < 90 m are not well captured in GRWL^2,12^, we first performed a comparison among the extracted river lengths based on the GRWL dataset, the HydroSHEDs dataset and our dataset (BGD) (Table 3). Then, a qualitative comparison was conducted with respect to the spatial distribution of river widths (Fig. 5) and the degree of detail of river extraction (Fig. 6). Finally, the accuracy of the extracted river widths was quantitatively evaluated with *in-situ* measured hydrological data from 58 cross-sections (Fig. 7, Supplementary File 1 Table 2, Supplementary File 1 Table 4 available at Figshare).

**Table 3:** Comparison of the river lengths (km) in the HydroSHEDs dataset, GRWL dataset and BGD.

River systems	HydroSHEDs			GRWL		BGD	
	order ≥ 3	order ≥ 4	order ≥ 5	all rivers	proportion of ≥ 4^th^-order rivers in HydroSHEDs/%	connected rivers	proportion of ≥ 4^th^-order rivers in HydroSHEDs/%
Yellow River	12558	6852	3571	4725	69.0	8194	119.6
Jinsha River	12142	7191	3589	6300	87.6	7518	104.5
Yalong River	8798	3390	1606	3313	97.7	3376	99.6
Lantsang River	4306	2520	1678	2750	109.2	2061	81.8
Nu River	6472	3577	1900	3194	89.3	2224	62.2
Yarlung Zangbo River	15812	8453	4447	6986	82.6	5622	66.5
Sum	60088	31983	16789	27269	85.3	28994	90.7

### Comparison of the extracted river lengths and the lengths in existing databases

The river lengths included in our dataset were compared to those in the HydroSHEDs dataset and GRWL dataset (Table 3). In general, the total lengths of the connected rivers provided in the BGD are 12205 and 2069 km longer than those of order 5 and higher rivers in the HydroSHEDs dataset and all rivers in the GRWL dataset, respectively (Table 3).

Particularly, for the Jinsha River and Yellow River, the connected river length in the BGD is even longer than that for ≥4th-order rivers in the HydroSHEDs dataset (Table 3). In addition, the connected river length in the BGD relative to that for ≥4th-order rivers in the HydroSHEDs dataset ranges from 62% to 120% (Table 3), which suggests that the BGD provides satisfactory coverage of medium- and high-order rivers in the study region.

### Qualitative validation with existing databases

#### Comparison with the GRWL database

The GRWL database, derived from Landsat imagery, provides global river width (>90 m) information under annual mean discharge^2^. Because different characterized discharges were used in the GRWL dataset and BGD, the absolute river widths of the two datasets were not compared. Therefore, two validations were performed: (1) comparison of the river width distribution under two characterized discharges (Fig. 5) and (2) qualitative evaluation by superimposing the BGD and GRWL datasets (Fig. 6).

The overall variation trends of river widths under bankfull and mean annual discharge conditions were relatively similar (Fig. 5). However, the peaks for the YLR exhibited large differences. For instance, the width peaks of the YLR were concentrated in the ranges of 40–70 m and 90–120 m for the BGD and GRWL datasets (Fig. 5), respectively, potentially because 90 m-resolution Landsat images were used for the GRWL dataset, and many rivers with widths <90 m were ignored. In addition, the width frequency peaks were more concentrated for the BGD than for the GRWL dataset in the three river basins (Fig. 5).

The extracted river surfaces from the BGD are more informative than those from the GRWL dataset. For the three northeastern river basins with river surfaces derived from Sentinel-2 imagery (YR, YLR, and JSR), the connected rivers in the BGD cover more than 98% of the GRWL river network, with only a few exceptions located in the YLR (Fig. 6a). For the southern three river basins (NR, LCR, and YLZBR), the richness of information for the GRWL dataset and BGD is comparable. The better representation of the BGD can be attributed to (1) the higher resolution of the RS imagery used for the YR, JSR and YLR, (2) the use of dynamic MNDWI thresholds for different river basins based on particular geological and geomorphological characteristics, and (3) more intense and accurate artificial editing.

#### Comparison with the global river network HydroRIVERs database

HydroRIVERs, developed by the Conservation Science Program of the World Wildlife Fund (WWF), is a database that provides a vectorized line network of rivers worldwide with a catchment area of at least 10 km^2^ or an average river flow of 0.1 m^3^ s^−1^ at a 15 arc-second resolution^18^. This database is part of HydroSHEDs^6^ and was derived from a 90 m DEM (Shuttle Elevation Derivatives at Multiple Scales 3, SRTM3). Horton-Strahler river orders were assigned to each river reach. The rivers in our study region are categorized as Horton-Strahler orders 1 to 8.

By comparing the surfaces of both connected and disconnected rivers extracted in this research and the vectorized river networks of HydroRIVERs, we evaluated the spatial distribution of rivers and the degree of detail of river extraction in this area (Fig. 6b). Generally, the river networks (order ≥ 3) in the HydroRIVERs dataset are richer than those in the BGD proposed in this research. For the YR, YLR and JSR, the extraction results (including both connected and disconnected rivers) cover most of the order 3 rivers (except for very few boundaries located in the northern part of the YR Basin) and almost all of the order 4–8 rivers of HydroRIVERs (Fig. 6b). This difference can be attributed to the high-resolution (10 m) Sentinel-2 imagery used in these watersheds. For the river surface derived from Landsat 5/7/8 (NR, LCR, and YLZBR), the coverage of the BGD is slightly smaller than that of HydroRIVERs (order ≥3) (Fig. 6b), which can be attributed to the relatively low RS image resolution used (30 m).

### Quantitative validation with *in-situ* measured data

Qualitative trend and spatial coverage comparisons are not enough to verify the performance of the above methods. It is also necessary to make a quantitative comparison based on measured data. The *in-situ* bankfull river widths measured at 58 cross-sections located at national hydrological stations (including 35 river widths that were not used for imagery selection; Supplementary File 1 Table 2 and Supplementary File 1 Table 4 available at Figshare) were used to quantitatively evaluate the river widths extracted from RS imagery. At each cross-section, we compared the *in-situ* measured river width to the average of the three spatially closest river width values obtained from the extracted river surface in our dataset. In general, the extracted river width results are satisfactory (Fig. 7); the absolute errors are within −16 m to 26.3 m, and at nearly all of the cross-sections, the relative errors between the measured and extracted widths are less than 20% (Supplementary File 1 Table 2 and Supplementary File 1 Table 4 available at Figshare). Two exceptions are as follows: Xiaqiaotou Station (100.050°E, 27.183°N), located in an order 4 stream in the upper JSR Basin, is associated with a relative error of −20.1%, and Yangbajing Station (90.546°E, 30.078°N), located in the secondary tributary of the YLZBR, is associated with a relative error of 28.8%. The *R*^2^, *RMSE* (root mean square error), and *MBE* (mean bias error) results are 0.958, 9.312 m, and 0.227 m, respectively (Fig. 7). The *RMSE* is calculated within one pixel, and the overall river width is slightly overestimated. We applied validation using cross-sectional samples with river widths of 23.3 m to 439 m, values that provided high representativeness for rivers of different types from single-thread to multithread in the QTP region. The Sentinel-2 and Landsat scenes were sampled at times that, in general, matched BD timing, which resulted in the satisfactory accuracy of the extracted bankfull river widths.

### Error analysis

Sources of error in the dataset are as follows. (1) The estimations of the discharge and river width under bankfull conditions are partly uncertain at those cross-sections that do not have evident flood plains. Due to the incomplete coverage of the RS imagery, DD ranges were expanded to at most (1 ± 50%) × BD. Compared with the ± 50% change range for discharge, the corresponding range of variation in river width was relatively small (Supplementary File 1 Table 2 available at Figshare). In the −10% to −50% BD range, the range of bankfull river width was −0.1% to −39.5%, with a mean of −6.9%. In the +10% to +50% BD range, the range of bankfull river width was 0 to +10.7%, with a mean of +3.5% (Supplementary File 1 Table 2 available at Figshare). Despite this impact, the error related to the BD and river width is relatively small but not negligible. (2) Error may also result from using RS imagery with different resolutions. In general, river widths equal to or more than three times the image pixel size are relatively accurate^3^. In detail, for rivers extracted based on Sentinel-2 imagery, the extracted minimum river width is 10 m, and rivers with widths ≥30 m are reliably extracted^3^; for rivers extracted based on Landsat 5/7/8 imagery, the results for rivers with widths >90 m are reliable^2^. Disconnected rivers with widths <30 m and <90 m extracted from Sentinel-2 and Landsat 5/7/8, respectively, might be less accurate and can only be used as a reference in analyses of the bankfull river width distribution.

## Usage Notes

Bankfull river surface data (vector data in.shp format) can be opened in the ArcGIS platform. Connected and disconnected river surfaces are included. Users can measure the bankfull river width and bankfull river surface area at any river length interval, as the dataset proposed in this research include width and area information every 1 km along the connected river length. Other applications, such as hydrological routing models and analyses of the spatial distributions of fluvial geomorphology, can also be explored based on this original dataset.

The proposed database has some limitations:

1. The coverage of the Sentinel-2 imagery is not complete for the dates on which the bankfull conditions were met for the LCR, NR and YLZBR. Landsat 5/7/8 imagery was then chosen as a substitute, which resulted in the omission and discontinuity of some small rivers with widths <90 m.
2. We had to choose between the extraction of small rivers (width <90 m) and rivers with large widths under bankfull conditions. Based on Sentinel-2 imagery, more small rivers with widths <90 m could be extracted. However, this choice resulted in the incomplete coverage of Sentinel-2 imagery, and the BD variation range had to be expanded to satisfy the coverage requirements. The Landsat 5/7/8 imagery was sufficient for filling this gap and provided more complete coverage of the NR, LCR and YLZBR; however, the data for rivers with widths <90 m was not robust in all cases. To maximize the consideration of bankfull conditions and maintain the strong physical meaning of our dataset, we neglected the extraction of small rivers with widths <90 m, and Landsat 5/7/8 imagery was used. Nevertheless, we believe that the BGD presented here provides the best current extraction results for bankfull river surfaces on the QTP.
3. According to the study of Elmi *et al*.^30^, region-based classification algorithms with high computational effort perform better than pixel-based methods for extracting water body. However, sometimes due to the high computational effort, pixel-based algorithms are preferred. Considering the accuracy and efficiency of water body extraction, in this study, we used the pixel-based Otsu dynamic thresholding method to extract water bodies. We also considered some complimentary measures, such as using different MNDWI thresholds for the contributing regions of different hydrological stations and the HydroSHEDs river networks to establish buffer zones, to reduce the influence of non-water background on river masks.
4. The implementation of the ARWE over the entire QTP demonstrates that our method has the potential to be employed in large areas and displays good applicability. In the future, we plan to update the Sentinel-2 images as soon as the newest *in-situ* measured hydrological data under bankfull conditions become available to us.

## Data Availability

The JavaScript algorithms used for cloud-free image composition and water surface extraction based on the GEE platform are provided in Supplementary File 2 at Figshare^29^. The MATLAB algorithms used for the generation of skeletonized river networks, control points and vertical lines perpendicular to river centerlines and the associated.xlsx files are provided in Supplementary File 3 at Figshare^29^.
